# The Role of Tumor pH in Breast Cancer Imaging: Biology, Diagnostic Applications, and Emerging Techniques

**DOI:** 10.3390/diagnostics16010076

**Published:** 2025-12-25

**Authors:** Dyutika Kantamneni, Saumya Gurbani, Mary Salvatore

**Affiliations:** 1 Department of Radiology, Jacobi Medical Center, Bronx, NY 10461, USA; 2 Department of Radiology and Imaging Sciences, Emory University, Atlanta, GA 30322, USA

**Keywords:** breast neoplasms, early detection of cancer, diagnostic imaging, mammography, tumor microenvironment, proton magnetic resonance spectroscopy, carbon-13 magnetic resonance spectroscopy, positron emission tomography computed tomography

## Abstract

Breast cancer screening, while vital for reducing mortality, faces significant limitations in sensitivity and specificity, particularly in dense breasts. Current modalities primarily detect anatomical changes, often missing biologically aggressive tumors at their earliest stages. The altered metabolism of cancer cells establishes a characteristic inverted pH gradient that drives tumor invasion, metastasis, and treatment resistance. This makes tumor acidity a compelling, functional biomarker for early detection. This review synthesizes the emerging role of pH as a diagnostic biomarker and provides a critical evaluation of advanced imaging techniques for its non-invasive or minimal measurement. We detail the biological underpinnings of tumor acidosis, emphasizing its regulation through glycolytic reprogramming and dysregulated proton transport. Our analysis encompasses a broad spectrum of pH-sensitive imaging modalities, including magnetic resonance methods such as Chemical Exchange Saturation Transfer (CEST) MRI for extracellular pH mapping and multi-nuclear Magnetic Resonance Spectroscopy (MRS) using ^1^H, ^31^P, and ^19^F nuclei to probe various cellular compartments. Furthermore, we examine hyperpolarized ^13^C MRI for real-time metabolic flux imaging, where metrics such as the lactate-to-pyruvate ratio demonstrate significant predictive value for treatment response. The review also assesses optical and photoacoustic imaging techniques, which offer high sensitivity but are often constrained to superficial tumors. Imaging tumor pH provides a powerful functional window into the earliest metabolic shifts in breast cancer, far preceding macroscopic anatomical changes. The ongoing development and evidence support the role of the pH-sensitive imaging techniques in diagnosis, lesion characterization, and therapy. Additionally, it holds promise for supplementing breast cancer screening by enabling earlier, more specific detection and personalized risk stratification, ultimately aiming to improve patient outcomes.

## 1. Introduction

Despite widespread adoption of breast cancer screening, tumors can still remain underdetected, contributing to continued morbidity and mortality [1]. The most common cancer among women worldwide is breast cancer, with 2.3 million new cases annually accounting for 30% of cancers in women with rising incidence rates [2,3]. To reduce mortality by early detection of cancer, the US Preventive Services Task Force (USPSTF) recommends biennial screening with digital mammography (DM) and digital breast tomosynthesis (DBT, also known as 3D-mammography) for women aged 40 to 74. Supplemental modalities such as breast ultrasonography, magnetic resonance imaging (MRI) and contrast enhanced mammography are advised for women with high lifetime risk based on familial and personal factors [4]. While patients with dense breasts are challenging to screen with DM and DBT, and there is ongoing research into the use of supplemental screening techniques such as MRI in these patients, per the USPSTF there is currently insufficient evidence to recommend this modality for screening. For diagnosis, additional modalities such as dynamic contrast enhanced MRI (DCE-MRI) which evaluates the angiogenic changes in malignancy, have shown increased specificity for the detection of breast cancer compared to non-malignant processes [5]. DCE-MRI can also be used to monitor treatment response in patients undergoing neoadjuvant chemotherapy, though differentiating residual disease from treatment changes remains challenging [6]. 

Not all women with high mammographic breast density (MBD) will develop breast cancer, and some women with low MBD will, which is why another marker for breast cancer risk is needed [7,8]. All of the aforementioned imaging modalities rely on identification of morphological changes in cancer, and as a result they have notable limitations: reduced sensitivity and specificity in dense breasts, risk of false positives and false negatives, and potential for overdiagnosis, all of which can lead to increased testing , invasive follow-up procedures, and psychological distress [4,9,10,11,12]. Moreover, by the time morphological abnormalities are visible, some tumors may already exhibit aggressive behaviour [13]. This underscores the need for screening approaches capable of detecting cancer earlier and with greater biological specificity, particularly in patients with dense breast tissue. One promising avenue is functional and metabolic imaging, such as targeting tumor pH. Cancer cells, unlike normal cells, exhibit altered metabolism to support intensive growth and turnover characterized by a more basic intracellular pH and more acidic extracellular pH due to the Warburg effect [14,15,16,17,18]. The Warburg effect is a well-known phenomenon in which tumors, even in the presence of sufficient oxygen, produce lactate through the glycolytic pathways rather than oxidative phosphorylation [18]. An acidic pH in breast cancer cells has been seen to contribute to increased invasive growth, migration, aggressiveness, metastasis, and poor prognosis, making pH a compelling biomarker for early breast cancer screening and detection [19,20,21,22,23,24,25].

Recent improvements in advanced imaging modalities can help improve our ability to detect breast cancer earlier by overcoming the limitations of traditional techniques, and may provide evidence for future improvements in screening and diagnostic guidelines, particularly for patients with dense breast tissue. This review synthesizes current evidence and emerging advances in pH-sensitive breast imaging, comparing these innovations with established modalities to evaluate their feasibility for earlier cancer detection.

## 2. Tumor pH Biology and Its Role in Breast Cancer

### 2.1. Why pH Is a Hallmark of Cancer?

Tumor pH is a defining metabolic hallmark of tumors, characterized by an inverted pH gradient due to an altered metabolism, a fundamental reversal from physiology that Koltai T termed “*the cancer pH paradigm*” [26,27,28,29,30]. Typically, extracellular pH (pHe) ranges from 7.3 and 7.4 while intracellular pH (pHi) ranges from 7.0 to 7.3 [31,32]. The reversal of this pHi/pHe gradient in cancer is a result of interplay between glycolytic reprogramming and dysregulated proton dynamics [18,33,34]. The Warburg effect, describing tumor cells’ increased aerobic glycolysis, drives lactate and proton (H^+^) overproduction; these acids are extruded via a Na^+^/H^+^ exchanger (NHE), the H^+^-lactate co-transporter, monocarboxylate transporters (MCTs), and the H+-ATPase (H^+^ pump), which lead to the secretion of H^+^ creating an acidic tumor microenvironment and an alkaline cytosolic environment [33,34]. The change in pHi promotes cell proliferation and cell survival by limiting apoptosis, which is associated with intracellular acidification [35,36,37,38,39,40]. Extracellular acidosis actively drives malignancy by promoting invasion (via MMP-9 activation), evasion of immune surveillance, and enhancing chemoresistance, among other effects [28,41,42,43,44,45,46,47,48]. In breast cancer, this pH inversion critically influences tumor aggressiveness, metastatic potential, and treatment response, while also offering opportunities for early detection strategies. Detecting tumor acidity through pH-sensitive imaging could identify cancers at their earliest metabolic stages, long before structural changes appear on traditional mammograms or DBT.

### 2.2. Acidic Shift in Breast Cancer

In cancer, there are multiple pathways that contribute to the regulation of pHe/pHi [49,50]. Cancer cells tend to have a higher alkaline pHi (>7.4) and lower acidic pHe (6.7–7.1) compared to normal cells [15]. This shift is not a passive occurrence but an active, orchestrated process driven by metabolic reprogramming and precise molecular regulation. In breast cancer cells, the acidity of the extracellular environment is attributed to multiple causes. The Warburg effect (aerobic glycolysis) is initiated by oncogenic signaling and a failure of energy-sensing checkpoints, particularly the loss of the LKB1-AMPK pathway, which allows cells with inefficient, acid-producing metabolism to evade growth arrest and continue proliferating [51,52]. Malignant tumors have increased tumor pHi than lower grade tumors [53]. The massive production of lactic acid and protons from glycolysis creates a critical need for acid extrusion. This is achieved through the coordinated upregulation of specific transporters on the plasma membrane or through phagosomes [54,55]. Monocarboxylate transporters MCT1 and MCT4 (SLC16A1, SLC16A3) are pivotal, co-exporting lactate alongside protons (H^+^) into the extracellular space, directly acidifying the tumor microenvironment [56,57,58]. Their expression and membrane localization are dependent on their association with the chaperone protein CD147, which is itself linked to poor prognosis [59,60,61]. Simultaneously, proton pumps, specifically the plasma membrane H^+^-ATPase, operate aggressively and uniformly across the cell surface to pump protons out, further contributing to the severe extracellular acidification [54,62]. Additionally, phagosomes can also contribute as they lead to increased leakage of protons [55,63,64].

To survive the acidic environment they are creating and to maintain the alkaline pHi necessary for cell proliferation, cancer cells concurrently activate acid-neutralizing import mechanisms. The Na^+^/H^+^ exchanger (NHE1) extrudes intracellular H+ in exchange for extracellular Na^+^, while the Na^+^, HCO_3_^−^ cotransporter NBCn1 (SLC4A7) imports bicarbonate ions, which buffer the cytosol [65,66,67]. Lowered cancer cell proliferation and reduced breast tumor growth is seen when the expression of NBCn1 is disrupted, but elevated levels are associated with shorter survival and increased metastasis [68,69]. In contrast, NHE1 is linked to promoting motility in ErbB2-positive breast cancer cells, stimulating the epithelial-to-mesenchymal transition (EMT) and contributing to chemotherapy resistance [65,66,70,71,72]. Additionally, a loss of the extracellular HCO_3_^−^ sensing protein receptor protein tyrosine phosphatase RPTPγ is observed in premalignant lesions of the breast. The upregulation or downregulation of these transporters is not random; it is directly driven by oncogenic pathways. For example, the tyrosine kinase ErbB2 (HER2), a key driver in certain breast cancer subtypes, enhances the expression and activity of both NHE1 and NBCn1 via the ERK-RSK signaling pathway. This direct link ensures that the pH regulatory machinery is integrated with the core proliferative signals of the cancer cell [73,74,75].

**Figure 1:**
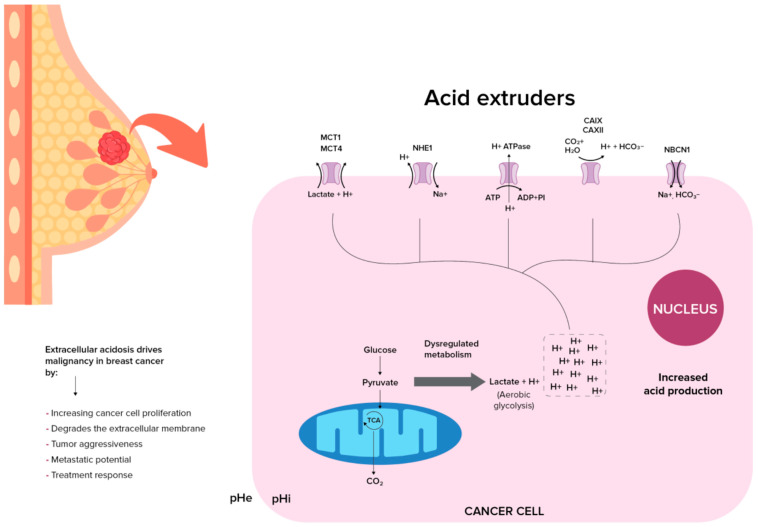
Acidic tumor microenvironment in breast cancer, resulting from dysregulated metabolism. The Warburg effect leads to increased proton (H+) and lactate production, causing extracellular acidification and intracellular alkalinization. Key transporters and enzymes contributing to this pH dysregulation include monocarboxylate transporters (MCT1, MCT4), Na^+^/H^+^ exchanger (NHE1), H^+^-ATPase, Na^+^-HCO_3_^−^ cotransporter (NBCn1), and carbonic anhydrases (CAIX, CAXII). pHe, extracellular pH; pHi, intracellular pH; TCA, tricarboxylic acid cycle; ATP, adenosine triphosphate; ADP, adenosine diphosphate.

The resulting inverted pH gradient is profoundly functional, promoting invasion and metastasis. Intracellular alkalinization protects the cancer cell from acid-induced damage and promotes proliferation [54,62]. Conversely, extracellular acidification remodels the tumor microenvironment by activating secreted enzymes, such as cathepsins, which degrade the extracellular matrix (ECM), thereby clearing a path for invasion. Furthermore, the acidic pHe impairs immune cell function, creating an immunosuppressive shield [76]. Thus, the coordinated activity of MCTs, H^+^-ATPases, NHE1, and NBCn1, all of which are regulated by oncogenic drivers, creates a permissive environment for tumor growth, invasion, and ultimately, poor patient outcomes (Figure 1).

## 3. Imaging Techniques for pH Detection in Breast Cancer

Due to changes in pH within the tumor, various imaging techniques are being evaluated to measure in vivo acidosis, including magnetic resonance-based methods (^1^H-MRS, ^31^P-MRS, ^19^F-MRS, and hyperpolarized ^13^C MRI), CEST imaging, optical/fluorescence imaging, photoacoustic imaging (PAI), and Fluorodeoxyglucose-18 positron emission tomography scan (FDG-PET). In this section, we review the imaging techniques listed above, in the order presented, and their application in breast cancer.

### 3.1. MR Spectroscopy Methods

Magnetic resonance spectroscopy (MRS) techniques offer the ability to derive metabolic information without the use of ionizing radiation [77,78]. Unlike standard MRI, which relies on the bulk signal of all protons, MRS techniques rely on the fact that different proton groups in molecules will resonate at slightly different frequencies in a magnetic field [79]. MRS techniques seek to identify and quantify different cellular chemicals based on the relative signals detected at these resonance offsets, providing quantitative insight into tissue metabolism in vivo [78,80]. MRS operates on the same scanner as an MRI but is uniquely designed to detect and quantify the concentrations of specific endogenous metabolites within living tissue [79,80]. By revealing these metabolic alterations, MRS can identify pathological processes even before any structural damage becomes visible [78]. However, the technique presents significant technical challenges, as it is highly sensitive to magnetic field imperfections, must detect molecules present in very low concentrations, and requires complex analysis to interpret the resulting spectral data [77,78].

a. **Proton** **Magnetic Resonance Spectroscopy (^1^H-MRS)**

^1^H-MRS represents a valuable approach for assessing tumor microenvironment parameters through detection of both endogenous metabolites and exogenous probes, leveraging the high natural abundance (99.98%) and superior sensitivity of the proton nucleus in human tissue [78,81]. This technique has demonstrated clinical utility in breast cancer characterization through detection of elevated choline compounds (tCho), which serve as a biomarker of increased membrane turnover in malignant tissues, with diagnostic sensitivity and specificity ranging from 71 to 80% [82,83,84]. More recently, attention has turned to lactate as a hypoxia-associated metabolite, with the study demonstrating that lactate can be detected in the human breast noninvasively using ^1^H-MRS [85].

However, conventional ^1^H-MRS faces challenges in breast tissue due to the high concentration of lipids in breast tissue. Lipid resonances can overlap with many other relevant chemical compounds, including choline. Techniques used elsewhere in the body, such as placement of saturation bands or masking of lipid-containing tissues, can be difficult to implement in the breast [86,87]. Double-quantum filtered (DQF) MRS methods have been developed to suppress these interfering lipid signals, enabling the specific detection of lactate and revealing significantly higher levels in grade III compared to grade II breast lesions, which supports its potential as a biomarker of tumor aggression [87,88,89,90,91,92,93].

For direct measurement of extracellular pH (pHe), exogenous imidazole-based probes such as (±)2-imidazole-1-yl-3-ethoxycarbonylpropionic acid (IEPA) and ISUCA have been developed, featuring pH-sensitive ^1^H resonances in the 7–9 ppm range, a spectral region relatively free from endogenous interferences [94,95,96,97,98,99]. These compounds enable ratiometric measurements through pH-dependent chemical shifts of their H-2 proton relative to internal reference protons, allowing concentration-independent pH assessment. Preclinical studies in orthotopic breast cancer models have successfully generated pHe maps using these approaches, revealing acidic regions within tumors that correlate with aggressive features [95,100]. While ^31^P MRS offers a more direct way to monitor phospholipid metabolites, energy metabolites, and intracellular pH levels, the increased spectral resolution at higher field strengths (7 T versus 1.5 T) has enabled distinct detection of phosphoethanolamine (PE) metabolites from phosphocholine (PC) metabolites in patient studies [101,102,103,104,105]. Clinical observations show altered metabolite ratios in breast cancer patients, with elevated PE resonances compared to PC resonances, which may partially explain the tCho levels detected. Furthermore, alterations in pH levels have been observed clinically through split Pi resonances, with calculations yielding pH values of 7.5 and 6.9 that align with literature reports [106,107].

However, the technique faces challenges, including relatively small pH-dependent chemical shifts (approximately 0.7 ppm over the physiological range) that require high magnetic field strengths (e.g., 3 T) for adequate resolution, and spatial resolution on the order of multiple millimeters that may be insufficient for characterizing heterogeneous tumor microenvironments [100]. Additionally, some imidazole-based probes exhibit buffering capacity that could potentially alter the pHe they are intended to measure, although recent developments in diimidazole compounds aim to address these limitations with enhanced sensitivity and reduced buffering capacity [94,108]. Despite these challenges, the technique’s compatibility with standard clinical MRI systems and its ability to simultaneously assess multiple metabolic parameters position it as a promising multiparametric approach for evaluating breast cancer.

b. **Phosphorus** **MRS (^31^P-MRS)**

**Figure 2:**
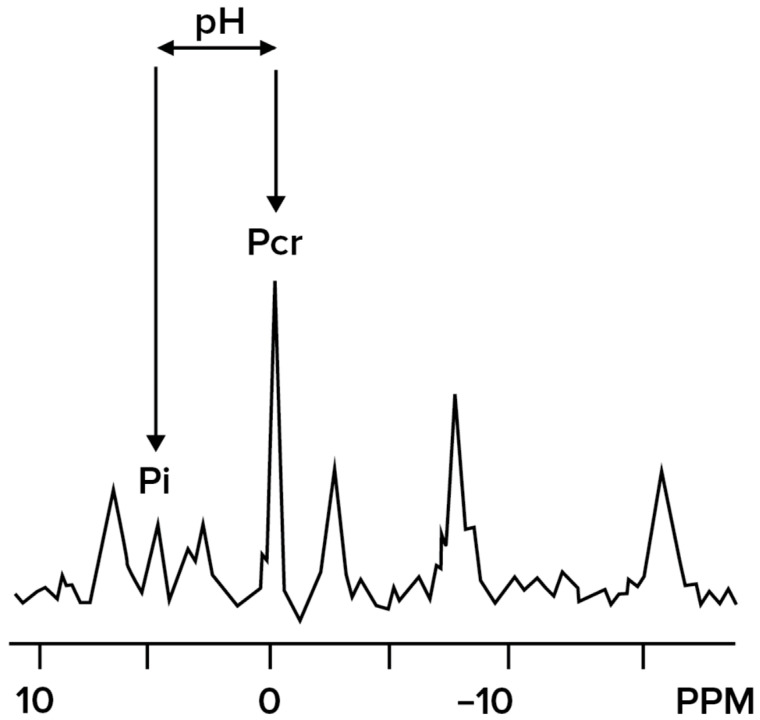
^31^P-MRS estimates pH from the relative change in chemical shift of inorganic phosphate (Pi), which changes with pH, relative to phosphocreatine (PCr), which remains constant.

Phosphate compounds play a central role in cellular energy metabolism; all energetic processes in human tissue cells involve phosphate-containing metabolites such as phosphocreatine (PCr), inorganic phosphate (Pi), and adenosine triphosphate (α-β-γ-ATP) [109]. Phosphorus MR Spectroscopy (^31^P-MRS) relies on the same phenomenon of difference in resonance frequencies of phosphorus when it is bound in different chemicals. ^31^P-MRS can measure intracellular pH by assessing the chemical shift difference between different phosphorus chemical compounds, with this difference changing in response to pH; this technique has been used to assess pH changes in multiple tissues and tumors [110]. Inorganic Pi is most affected by changes in pH, particularly in tumors, whereas PCr is used as a reference since it has a relatively stable resonance position across physiologic pH ranges [111,112,113] (Figure 2). Studies on 3 T MRI have shown an improved signal-to-noise ratio and spectral resolution in the human breast in vivo. Most studies have focused on the quantification of phosphorus metabolites in breast cancers as compared to normal tissue, with a focus on changes of these metabolites under chemotherapy, with an increase in pHi following treatment [114,115,116,117,118,119]. While MRS can be used to study the intracellular pH changes, which are the initial changes that occur in cancer cells, further studies have been conducted to assess the extracellular pH [120]. 31P MRS of 3-aminopropylphosphate (3-APP), a non-toxic, membrane-impermeant compound, is used to measure the extracellular pH of the tumor. It has been demonstrated to show changes in breast cancer cell lines [27]. A major benefit of ^31^P-MRS is the lack of need for water suppression to detect phosphorus metabolites, unlike in vivo metabolite detection with ^1^H-MRS [119]. Limitations of a ^31^P-MRS include lower sensitivity as Pi concentrations are generally lower than proton concentrations, and thus it is essential to use either slower or lower resolution techniques to acquire sufficiently high SNR for pHe quantification [110].

c. **Fluoride** **MRS (^19^F-MRS)**

Fluoride MR spectroscopy (^19^F-MRS) has emerged as a powerful technique for probing the tumor microenvironment, particularly extracellular pH, due to the near absence of endogenous fluorine background in biological tissues [121,122,123]. This lack of background signal allows for unambiguous detection of exogenous fluorinated probes, while the nucleus’s high gyromagnetic ratio and large chemical shift dispersion (~300 ppm) provide excellent spectral resolution [121]. The development of effective ^19^F-MRS pH indicators requires careful optimization of pharmacokinetic properties, including a pK_a_ within the physiological range, high sensitivity and specificity, low toxicity, efficient cellular delivery, and appropriate membrane permeability characteristics [120,122,123,124,125].

Several classes of fluorinated probes have been developed for pH sensing. Early approaches utilized fluorinated alanines, which demonstrated pH-dependent chemical shifts but were limited to perfused cell systems and were not applied in vivo [126,127,128,129]. The fluoroaniline sulfonamide ZK-150471 has shown particular promise, demonstrating a superior signal-to-noise ratio and pH sensitivity compared to ^31^P-MRS probes, such as 3-APP [120,130].

Recent innovations focus on “smart” contrast agents and nanoscale delivery systems [131,132,133]. PEGylated nanogels containing perfluorocarbons exhibit pH-dependent size changes, enabling indirect pH estimation through ^19^F signal variations [134,135,136,137,138,139]. Molecular switches acting as ratiometric probes eliminate the need for external references, while paramagnetic relaxation enhancement strategies using Mn^2+^ ions have improved signal intensity by over five-fold [135,137,140,141]. Liposil-encapsulated fluorinated ionic liquids represent another advancement, enabling multiplexed ^19^F MRI with enhanced relaxation properties [141].

Despite these advantages, ^19^F MRS faces challenges including probe instability, nonspecific accumulation due to hydrophobicity, and limited spatial resolution despite high field strengths [130,134]. While extracellular pH measurement is well-established, simultaneous assessment of intracellular pH remains challenging. Nevertheless, the continuous development of novel fluorinated compounds and encapsulation strategies positions ^19^F MRS as a valuable complementary approach to proton-based methods for characterizing tumor acidosis in breast cancer research and potential clinical applications [134,137].

### 3.2. Hyperpolarized Carbon-13 (^13^C-MRI)

Hyperpolarized carbon-13 (^13^C) magnetic resonance imaging (MRI) represents an advanced metabolic imaging technique that enables real-time, non-invasive assessment of tissue pH. The method enhances the signal of ^13^C-labeled compounds by cooling a ^13^C-enriched sample with a polarizing agent to near absolute zero in the presence of strong magnetic fields (3.35—7 T) for 2 h, and then transferring polarization via microwave irradiation to improve visualization of metabolic activity. However, this technique requires specialized equipment for polarization and rapid quality control, necessitating dedicated multinuclear MRI scanners with custom ^13^C coils. These technical and economic constraints have currently limited its widespread clinical implementation [142].

Two primary approaches facilitate pH quantification using hyperpolarized ^13^C MRI. The first method utilizes ^13^C-bicarbonate, which undergoes pH-dependent equilibrium with ^13^CO_2_ via carbonic anhydrase catalysis, enabling quantitative mapping of extracellular pH through signal intensity ratios. The second approach employs [1-^13^C] pyruvate, whose metabolic conversion to ^13^CO_2_ in highly aerobic tissues permits similar ratio-based calculations primarily reflecting intracellular pH. Since both compounds are endogenous metabolites, these techniques show significant potential for clinical translation in mapping pH distributions in human cancers [143].

In breast cancer applications, the hyperpolarized ^13^C-lactate signal has demonstrated utility as a potential biomarker for tumor grading, with detectable signals in triple-negative and high-grade tumors but absence in lower-grade malignancies [144,145]. The lactate-to-pyruvate ratio (Lac/Pyr) correlates strongly with HIF1α expression, linking metabolic activity to hypoxia and tumor volume, while also showing associations with lactate dehydrogenase A (LDHA) and monocarboxylate transporter 1 (SLC16A1/MCT1) expressions [146].

The technology shows particular promise for assessing treatment response, detecting metabolic alterations within 7–11 days post-treatment. Increases in Lac/Pyr ratio ≥ 20% following therapy have demonstrated predictive value for pathological complete response (pCR) [146,147]. However, data interpretation requires careful consideration, as the ^13^C-lactate signal reflects complex interactions involving substrate delivery, transporter expression, and enzymatic activity, rather than just lactate production. This complexity is evidenced by paradoxical observations such as increased lactate conversion following pazopanib treatment [148].

Recent technological innovations address limitations of conventional polarization methods. Novel approaches, such as Spin-Lock Induced Crossing with Signal Amplification by Reversible Exchange (SLIC-SABRE), enable the rapid generation of hyperpolarized [1-^13^C] pyruvate within minutes at a reduced cost, facilitating high-throughput studies. Applications in transgenic breast cancer models have successfully identified elevated lactate metabolism and metabolic subcompartments corresponding to histological profiles, demonstrating potential for overcoming current implementation barriers [149].

While challenges remain regarding technical complexity, cost, and data interpretation, ongoing advancements in hyperpolarized ^13^C MRI position it as a transformative approach for non-invasive pH monitoring in breast cancer management. The capability to track spatial and temporal pH dynamics provides unique insights into tumor heterogeneity and treatment response, offering significant potential for advancing personalized cancer care [144].

### 3.3. Chemical Exchange Saturation Transfer (CEST) MRI

**Figure 3:**
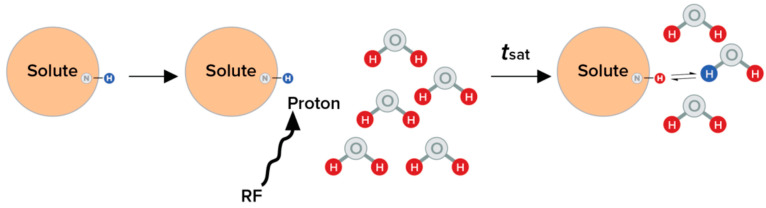
Principle of Chemical exchange saturation transfer (CEST): the protons on the solute, once saturated, are exchangeable (blue) at a specific frequency, and this saturation is transferred to water following a period of t_sat_. RF, radiofrequency frequency; tsat, time required for saturation to be transferred to water.

Chemical exchange saturation transfer (CEST) imaging refers to a method for measuring low-concentration solutes through the phenomenon of magnetization transfer (MT). In MT, a specific proton group (e.g., amides) is stimulated at its resonance frequency using radiofrequency (RF) pulses, saturating its magnetization. This magnetization is transferred to the surrounding water due to the exchange of the excited protons with protons in bulk water. Repeated cycles of stimulation and transfer will lead to a build-up of signal in bulk water, which cannot be saturated, given its concentration is orders of magnitude higher than the stimulated proton groups. This increased signal can then be more easily detected [150,151,152,153,154]. The chemical exchange of saturated protons with protons of water is pH-dependent, and therefore, measurement of the rate of transfer can be used to estimate tissue pH [155] (Figure 3). CEST imaging has proven effective in detecting acidic pH of breast tumors and revealing their metastatic potential, as the CEST effect diminishes at higher, more basic pH levels due to an excessively rapid exchange rate. CEST is broadly categorized into two types: endogenous and exogenous CEST [156,157,158,159].

a. **Endogenous** **CEST:**

This approach focuses on the stimulation of chemical groups endogenously found in tissues. The most established method is amide proton Transfer(APT) imaging, which focuses on amide protons (proteins and peptides) [159]. The exchange rate between the amide protons and water decreases with decreasing pH [155,160,161,162]. Since cancer cells exhibit higher intracellular protein concentrations due to rapid turnover, the APT signal is often increased [163,164,165]. Clinically, APT has demonstrated high diagnostic performance, distinguishing between benign and malignant breast lesions with an AUC as high as 0.959 [166,167]. Its signal shows a positive correlation with tumor stage and Ki-67 proliferation index, and decreases following effective neoadjuvant therapy [168]. A current limitation is that it has mostly been studied for characterizing larger lesions compared to smaller ones.

Other endogenous CEST methods target different protons, including hydroxyl groups (e.g., glycoCEST and gagCEST, used in humans to distinguish tumors from surrounding tissue) and the amine groups of glutamate (gluCEST) [108,169,170]. While GluCEST has historically been used in the brain, recent work has shown promise in mouse models for monitoring early response to glutaminase inhibitors in triple-negative breast cancer [171]. Its clinical translation for breast cancers has been somewhat limited as it relies on high magnetic field strengths such as 7 T, which are not typically used in breast imaging [172].

b. **Exogenous** **CEST:**

This method utilizes administered CEST contrast agents to specifically map the extracellular pH (pHe) of the tumor microenvironment [173]. It employs FDA-approved, non-ionic iodinated X-ray contrast agents known for high water solubility, low toxicity, and pH dependence, such as Iopromide, Ioversol [174,175,176]. Recent work in mouse models using iopamidol CEST found a correlation between breast tumor acidity and metastasis potential, and the relation with cancer with varying metabolic activity and HIF-1A expression [156,157,177]. A key advance is the development of acidoCEST MRI, a technique that leverages the differences in CEST effects of its two amide groups at varying pH levels. A ratio of the magnitude of these two CEST effects was found to be highly linearly correlated with pH in breast cancer [176]. This enables a ratiometric analysis method that calculates pHe independently of the agent’s concentration, a significant advantage for use in human subjects [174,178,179].

However, exogenous CEST faces challenges. Many pioneering studies have been conducted at 7 T, which is not widely available even at academic institutions, and validation on clinical 3 T systems is therefore needed. The accurate measurement range is typically limited to pH < 7.2, and while many tumor microenvironments are at pH < 7.4, the technique remains sensitive to confounding variables like temperature, pH, contrast agent, and magnetic field (B0/B1) inhomogeneity, necessitating careful experimental control [180,181].

### 3.4. Optical and Fluorescent Probes for Tumor pH Detection

Optical imaging represents a powerful approach for detecting the acidic tumor microenvironment, leveraging light-based technologies to provide accurate, real-time data on biochemical properties, including extracellular pH (pHe) [182,183]. These methods are particularly valuable for their high sensitivity and versatility, though their application is primarily limited to superficial or accessible tumors [106,184,185,186,187]. A key advantage of these approaches is their capacity for ratiometric measurements, where the ratio of signals at different emission or excitation wavelengths enables pH quantification independent of probe concentration, photobleaching, or tissue depth variations. This capability for concentration-independent measurement significantly enhances accuracy and reliability in pH detection [188].

For in vivo applications, the near-infrared (NIR) range (650–900 nm) is particularly valuable due to reduced light absorption and scattering by biological tissues, allowing penetration to deeper depths of the tissue of up to 1 cm. Several optical techniques have been developed for pH sensing, each with distinct characteristics [108,176]. Optical sensors such as Optical coherence tomography (OCT) and photoacoustic imaging (PAI) are a non-invasive technique for detecting tumors [189].

OCT employs non-invasive light-based imaging to generate high-resolution, micrometer-scale images of tissue architecture. OCT takes advantage of the high spatial resolution of near-infrared light to detect micrometer-level reflectances in tissue, an optical analogue of ultrasound [190]. OCT has demonstrated particular utility in identifying early-stage breast malignancies that evade detection by conventional imaging modalities. Clinical investigations have validated OCT’s diagnostic capability, reporting sensitivity of 93% and specificity of 85% in distinguishing cancerous breast tissue, establishing its potential as an effective tool for early cancer detection [191]. Fluorescence imaging utilizes pH-sensitive dyes that alter their emission properties in response to changes in acidity. Optical coherence tomography (OCT), although not a direct pH sensor, provides high-resolution structural imaging that complements pH measurements. The development of various probes has been crucial for advancing optical pH detection, ranging from visible-light probes like seminaphthorhodafluor-1 (SNARF-1) for preclinical window chamber models to NIR fluorescent probes such as modified rhodamine bound to dextran (Dex-Me-pEPPR) and activatable nanoparticles that turn on fluorescence in acidic conditions [192,193]. The pH can be measured independently of concentration by assessing the ratio of fluorescence signals at different emission wavelengths or at different fluorescence lifetimes [21,184,185,186,192].

PAI has emerged as a particularly promising modality for deeper tissue pH sensing, utilizing probes designed with pH-dependent and pH-independent absorption peaks [182]. To overcome the depth limitations of optical methods like photoacoustic imaging (PAI) has emerged as a modality for deeper tissue pH sensing. PAI is a hybrid modality that combines optical imaging with ultrasound detection, providing high spatial and temporal resolution for imaging structures several centimeters deep [194,195]. This technique can identify tumors as small as 1–2 mm with a sensitivity of 91%. Systems including albumin-based nanoparticles with benzo-α-phenoxazine and IR825, or polymer nanoparticles containing SNARF-5F, enable pH quantification through ratioing of optoacoustic signals at different excitation wavelengths [196]. Despite their considerable promise, the clinical translation of optical and photoacoustic pH-sensing for broad breast cancer screening faces hurdles. The primary limitation remains depth penetration, which restricts these methods to superficial or accessible tumors [106,196]. Furthermore, challenges related to biocompatibility and probe stability, along with addressing the complexities of absolute pH quantification due to nonlinear response curves, must be addressed [108]. Consequently, these technologies are currently best suited for specialized applications such as intraoperative guidance or endoscopic procedures, where their high sensitivity and capacity for spatial pH mapping can be fully leveraged [108,194,195,196,197].

### 3.5. FDG-PET

18F-fluorodeoxyglucose (FDG) PET/CT is widely used in oncology for diagnosis, staging, and surveillance [198]. As a non-invasive measure of glycolytic activity, FDG PET reflects the Warburg effect and enhanced reliance on aerobic glycolysis, which is observed in many cancers [199,200]. In breast cancer, higher FDG uptake correlates with increased concentrations of glycolytic metabolites, including lactate (*p* = 0.001) and L-acetylcarnitine (*p* = 2 × 10^−4^), which contribute to the acidic tumor microenvironment [200]. Because tumor acidosis arises largely from elevated glycolysis, regions of high FDG uptake correspond to areas of increased acidity, making FDG-PET a practical surrogate for visualizing tumor pH and assessing metabolic aggressiveness.

To more directly image tumor acidity, pH-low insertion peptides (pHLIPs) labeled with 89Zr have been developed as PET tracers that target acidic environments independently of molecular surface markers [201]. 89-Zr-DFO-Cys-Var3 is a PET probe that demonstrates uniform tumor staining and highlights acidified zones at the tumor–stroma interface. pHLIP uptake extends beyond hypoxic regions to stromal components, including metabolically active tumor-associated macrophages (TAM), which contribute to T-cell suppression [202,203,204]. Because acidosis promotes tumor progression, drug resistance, and immune dysfunction, imaging probes that map acidity may help predict and monitor responses to immunotherapy. Although pHLIPs have slower pharmacokinetics, the long half-life of 89Zr enables multi-day imaging, supporting their potential for clinical translation [201].

Beyond zirconium-labeled constructs, alternative radionuclides and chelation strategies have been explored to optimize pHLIP-based PET imaging. Both 18F and 64Cu can label NOTA- and NO_2_A-modified pHLIP peptides, with 18F offering broad availability and cost-effectiveness, while 64Cu provides a longer half-life, allowing for extended imaging windows. In 4T1 breast cancer models, negatively charged NOTA-conjugated variants clear rapidly via hepatobiliary and renal pathways, thereby limiting tumor retention. In contrast, neutrally charged NO2A-cys constructs exhibit slower blood clearance, improved tumor perfusion, and sustained trapping within acidic tumor regions. Among these, Var3 derivatives 64Cu-NO2A-cysVar3 and 18F-AlF-NO2A-cysVar3 exhibit the highest tumor uptake and tumor-to-background ratios across multiple models [205].

In summary, while FDG-PET does not directly measure pH, it provides a clinically accessible surrogate for regions of tumor acidosis in breast cancer, reflecting glycolytic activity and associated extracellular acidification. Together with pHLIP-based PET tracers that directly target acidic environments, these imaging approaches offer complementary strategies for visualizing tumor metabolism and pH, aiding in tumor characterization, prognostication, and potentially guiding therapy.

## 4. Discussion

This comprehensive review establishes tumor acidosis as a fundamental, targetable hallmark of breast cancer with profound implications for early detection. The inverted pH gradient, characterized by alkaline intracellular and acidic extracellular environments, is not merely a metabolic by product but an active driver of tumor progression, invasion, and treatment resistance. The central conclusion of this analysis is that pH-sensitive imaging represents a paradigm shift in breast cancer screening, moving beyond anatomical assessment to a functional and metabolic characterization.

The primary conclusion is that multiple imaging modalities have now matured to the point of providing reliable pH measurement [Table 1]. Techniques such as CEST MRI, particularly the exogenous acidoCEST approach, and advanced MRS methods (^19^F, ^31^P) offer clinically feasible pathways to quantify extracellular and intracellular pH with increasing precision. Hyperpolarized ^13^C MRI, despite its current technical complexity, provides unprecedented insight into real-time metabolic flux, with the lactate-to-pyruvate ratio emerging as a powerful predictive biomarker for treatment response. We included studies that demonstrated statistically significant pH-related findings, and readers are referred to the individual publications for detailed statistical methodologies and any correction procedures applied.

The relevance of these findings to the field is substantial and multifaceted. First, pH imaging addresses critical limitations of current screening and diagnostic techniques that rely on structural and morphological changes in tissue for differentiating malignancy from non-malignant processes, particularly in dense breasts, where structural modalities underperform. By detecting metabolic alterations that precede the formation of macroscopic tumors, these techniques can enable earlier cancer detection. Second, the ability to stratify tumors based on their pH profile and metabolic activity offers new avenues for personalized risk assessment and treatment selection, potentially identifying aggressive tumors that warrant more intensive management while sparing patients with indolent disease from overtreatment.

The translation of these technologies into routine clinical practice represents the next frontier. Although current pH Imaging techniques have been well studied and risk stratification and treatment monitoring, there remain limitations that preclude their use as broad screening tools for breast cancer screening. In particular, challenges in reproducibility, protocol standardization, cost, scanner availability, and validation across diverse patient populations must be addressed before large-scale clinical implementation is feasible. Importantly, emerging evidence linking pH-related imaging biomarkers such as hyperpolarized ^13^C lactate signals, elevated lactate on proton MRS, and higher endogenous CEST contrast to tumor stage and grade underscores the potential clinical value of these techniques once standardized and validated. Nevertheless, the convergence of growing biological understanding of tumor acidosis with rapid imaging innovation positions pH-based screening as a transformative approach. Future efforts should focus on integrating these functional techniques with existing structural modalities, developing standardized protocols, and validating pH thresholds that reliably predict clinical outcomes. With continued refinement and validation, pH-sensitive imaging holds the promise to revolutionize breast cancer detection by capturing disease at its earliest metabolic stages, ultimately improving personalized risk assessment, guiding timely intervention, and reducing mortality.

## Figures and Tables

**Table 1:** Diagnostic Imaging techniques for pH detection for breast cancer screening.

Technique	Primary Measure	Key Strength	Key Finding	Main Limitation	Typical Acquisition Times
**Techniques to evaluate pH**					
**^1^ H MRS (Conventional)**	Total Choline (tCho) concentration	High endogenous concentration; widely available on standard clinical MRI systems	71–80% sensitivity/specificity for malignant lesion detection	Indirectly measures pH; lipid signal contamination	5–10 min for single voxel spectroscopy [206] 10–15 min for multivoxel/2D [207]
**^1^ H MRS (DQF)**	Lactate concentration	Specific lactate detection by suppressing lipid signals	Higher lactate in grade III vs. grade II lesions; links to hypoxia	50% inherent signal loss; challenging for small lesions	10–12 min [93,208]
**^1^ H MRS** **(Exogenous Probes)**	Chemical shift of probe’s H-2 proton	Ratiometric, concentration-independent pHe measurement	Successful pHe mapping in preclinical models reveals acidic regions	Small chemical shift range (~0.7 ppm); may alter native pHe	~20–55 min [95,98]
**^31^ P MRS**	Chemical shift difference of inorganic phosphate compared to pH-independent phosphates I	Direct measurement of intracellular pH; monitors energy metabolism	Resolves multiple pH compartments via Pi splitting	Low endogenous concentration; poor spatial/temporal resolution	~25 min [118,209]
**^19^ F MRS**	Chemical shift of exogenous ^19^F probe	Negligible biological background; large chemical shift dispersion	Superior SNR vs. ^31^P MRS; enables specific pHe mapping	Requires exogenous probes; limited clinical translation	~5–20 min [210]
**Hyperpolarized ^13^C MRI**	Lac/Pyr or H^13^CO_3_^−^/^13^CO_2_ ratio	>10,000× signal enhancement for real-time metabolic flux	Lac/Pyr increase ≥20% predicts pCR post-treatment	Extreme cost/technical complexity; short signal lifetime	~3 min [211]
**CEST MRI** **(Endogenous)**	Amide proton transfer (APT) effect	No contrast agent needed; correlates with tumor aggression	High AUC (~0.96) for malignancy; tracks therapy response	Confounded by multiple factors; less sensitive in small lesions	2–13 min [212]
**CEST MRI** **(Exogenous)**	Chemical exchange of iodinated agents	Ratiometric, concentration-independent pHe measurement	Revealed “pH-neutral” tumors; quantitative pHe mapping	Limited to acidic range (pH < 7.2); requires high field strength (e.g., 7 T)	~30 min [156,176]
**Fluorescence** **Imaging**	Fluorescence intensity ratio	High sensitivity; real-time ratiometric quantification	Direct correlation between low pHe and high tumor invasion	Limited to superficial tumors (<1 cm depth)	Seconds to minutes [213,214]
**Photoacoustic Imaging**	Optoacoustic signal ratio	Deeper penetration than pure optical; combines optical/ultrasound	91% sensitivity for lesions 1–2 mm; quantifies tumor pH	Nonlinear response curves; probe biocompatibility issues	Seconds [215]
**Additional imaging modalities used in breast cancer that do not evaluate pH**					
**FDG-PET**	Glucose uptake as a surrogate for glycolytic activity	Widely available, non-invasive, clinically validated for staging and response assessment	Higher FDG uptake correlates with elevated glycolytic metabolites (lactate, L-acetylcarnitine), which are associated with extracellular acidification	Indirect measure of pH; cannot distinguish causes of high glucose flux (proliferation, inflammation, hypoxia)	50–70 min [216]
**^89^ Zr-pHLIP PET**	Direct binding of pHLIP peptides to acidic extracellular pH	Direct visualization of acidic TME; identifies stromal acidity and TAM-rich regions	Uniform tumor staining; highlights acidified zones beyond hypoxia, including immunosuppressive stromal regions	Slow pharmacokinetics; requires long-lived isotopes	Multiday imaging (24–72 h post-injection [201])
**^18^ F-AlF-NO_2_A-cysVar3/^64^Cu-NO_2_A-cysVar3 PET**	Direct pH-dependent peptide insertion	Practical radiosynthesis (especially ^18^F-AlF); improved tumor retention; higher T:B ratios	Neutral NO_2_A-cysVar3 variants show sustained trapping in acidic TME and superior contrast across breast, prostate, melanoma, and brain tumors	Some variants show hepatobiliary clearance; optimization still ongoing	Multi-day imaging [205] (24–72 h post-injection)
**Dynamic Contrast-Enhanced MRI**	Gadolinium uptake kinetics	Highest reported sensitivity for breast cancer diagnosis	Clinical gold standard for lesion detection and characterization	Requires contrast injection; high cost; not pH-specific	~5–7 min [217]
**Optical** **Coherence Tomography**	Tissue microstructure changes	High spatial resolution (~micrometers); non-invasive	93% sensitivity/85% specificity for cancerous tissue	Does not directly measure pH; structural context only	5 s per image [218]

## Data Availability

No new data were created or analyzed in this study. Data sharing is not applicable to this article.

## Abbreviations

The following abbreviations are used in this manuscript:

AMPK	AMP-Activated Protein Kinase
AUC	Area Under the Curve
DBT	Digital Breast Tomosynthesis
DFO	Desferrioxamine
DM	Digital Mammography
DQF	Double-Quantum Filtered
ECM	Extracellular Matrix
EMT	Epithelial-to-Mesenchymal Transition
ERK	Extracellular Signal-Regulated Kinase
FDG	Fluorodeoxyglucose
FDG-PET	Fluorodeoxyglucose Positron Emission Tomography
gluCEST	Glutamate Chemical Exchange Saturation Transfer
glycoCEST	Glycogen Chemical Exchange Saturation Transfer
gagCEST	Glycosaminoglycan Chemical Exchange Saturation Transfer
HER2	Human Epidermal Growth Factor Receptor 2
HIF-1α	Hypoxia-Inducible Factor 1 alpha
LKB1	Liver Kinase B1
LDHA	Lactate Dehydrogenase A
MMP-9	Matrix Metalloproteinase 9
NIR	Near-Infrared
NOTA	1,4,7-Triazacyclononane-1,4,7-triacetic Acid
NO_2_A	1,4,7-Triazacyclononane-1,4-diacetic Acid
OCT	Optical Coherence Tomography
PC	Phosphocholine
pCR	Pathologic Complete Response
PE	Phosphoethanolamine
PET-CT	Positron Emission Tomography–Computed Tomography
pHLIP	pH-Low Insertion Peptide
RSK	Ribosomal S6 Kinase
SLC16A1	Solute Carrier Family 16 Member 1
SLC16A3	Solute Carrier Family 16 Member 3
SLC4A7	Solute Carrier Family 4 Member 7
SNARF-1	Seminaphthorhodafluor-1
SNARF-5F	Seminaphthorhodafluor-5F
SNR	Signal-to-Noise Ratio
TAM	Tumor-Associated Macrophage
tCho	Total Choline
USPSTF	United States Preventive Services Task Force
